# The Chestnut Technique: A Novel Approach to Enhancing Implant Stability in Breast Augmentation

**DOI:** 10.1007/s00266-025-05062-w

**Published:** 2025-07-07

**Authors:** Mert Demirel, Mert Ersan

**Affiliations:** 1 Kadikoy, Istanbul, Turkey; 2 Plastic, Reconstructive and Aesthetic Surgery Department, Faculty of Medicine, Yeditepe University Kozyatagi Hospital, Icerenkoy Mahallesi, Yeditepe University, Hastahane Sokak 34752, Atasehir, Istanbul, Turkey

**Keywords:** Breast implants, Breast implantation, Mammaplasty, Breast, Pectoralis muscles, Postoperative complications

## Abstract

**Background:**

Despite advancements in implant technology and surgical techniques, achieving long-term implant stability in breast augmentation remains challenging, particularly in cases where enhanced soft tissue support is required.

**Objective:**

The chestnut technique was developed to enhance implant stability and address soft tissue support-related complications by incorporating a structurally supportive soft tissue framework. This study aims to describe the surgical steps of the chestnut technique and evaluate patient-reported outcomes following its application.

**Method:**

This retrospective study included 60 female patients who underwent primary breast augmentation between April 2020 and November 2024. Implants were placed in a customized subpectoral pocket, ensuring superior and inferior muscle and fascia coverage with pectoral fascia support at the midsection. Patient satisfaction was assessed using the BREAST-Q module preoperatively and at 1 year postoperatively.

**Results:**

The mean follow-up period was 31.8 months (range: 12–56 months). No cases of implant malposition, rippling, or animation deformity were observed. Minor complications occurred in 5% of patients and were successfully managed with conservative treatment. The mean BREAST-Q satisfaction score significantly increased from 23.44 ± 10.20 preoperatively to 84.46 ± 9.90 at 1 year, with similar improvements in psychosocial and sexual well-being (*p* < 0.001).

**Conclusion:**

The chestnut technique provides a safe and effective approach to primary breast augmentation, particularly in cases where enhanced soft tissue support is desired. By optimizing implant stability and minimizing displacement-related complications, it serves as a reliable alternative to conventional methods while ensuring predictable and aesthetically favorable outcomes.

**Level of Evidence IV:**

This journal requires that authors assign a level of evidence to each article. For a full description of these Evidence-Based Medicine ratings, please refer to the Table of Contents or the online Instructions to Authors www.springer.com/0026.

## Objective

Breast augmentation is one of the most frequently performed aesthetic surgical procedures worldwide, with steadily increasing demand due to both functional and cosmetic motivations [1]. Beyond its aesthetic benefits, the procedure has been shown to significantly enhance patient satisfaction and psychosocial well-being, reinforcing its role as a transformative intervention in plastic surgery [2–4].

Over the past decades, advancements in implant technology and surgical techniques have led to improved aesthetic outcomes and reduced complications such as capsular contracture, infection, and implant malposition [5–7].

Despite these advancements, implant stability remains a challenge, particularly in terms of soft tissue support at the inferior pole. Traditional augmentation techniques—including dual-plane and subfascial approaches—often fail to provide sufficient structural support, leading to complications such as implant displacement, bottoming-out, double-bubble deformity, rippling, animation deformity, and asymmetry [6–11].

To address these limitations, we developed the chestnut technique, a novel approach designed to enhance implant stability and mitigate soft tissue support-related complications by integrating a caudal and cranial flap system.

This retrospective study aims to describe the surgical steps of the chestnut technique and evaluate patient-reported outcomes following its application. Our findings contribute to the evolving discourse on breast augmentation by presenting a refined surgical approach.

## Methods

### Study Design and Patient Selection

This retrospective study included 60 female patients, aged 18–59 years, who underwent primary breast augmentation between April 2020 and November 2024. All participants were classified as ASA I or II and underwent submuscular implant placement for aesthetic enhancement.

Patients scheduled for subglandular or subfascial augmentation, those requiring revision surgery, or individuals with a history of breast disease were excluded. Additionally, patients with a BIRADS score above 3 on ultrasound or mammography within the past year were not included. Patients with less than 1 year of postoperative follow-up and those unable to complete the 1-year satisfaction survey were also excluded.

### Preoperative Evaluation

A comprehensive preoperative evaluation was conducted to assess key anatomical features relevant to implant selection and surgical planning. The inframammary fold (IMF) was carefully analyzed for its localization, prominence, symmetry, and relationship with the pectoralis major and rectus abdominis muscles [12, 13]. Breast tissue thickness was evaluated at the inferior and medial poles, and a quadrant-based parenchymal assessment was performed to identify any structural variations. Any palpable masses detected during the examination were documented.

Key anthropometric measurements, including nipple position, distance to the IMF, and distance to the sternal notch, were recorded to assess potential asymmetries. Any discrepancies in these parameters were noted for consideration in surgical planning.

Preoperative planning involved a detailed assessment of the breast footprint, tissue quality, thoracic wall asymmetries, spinal alignment, overall skeletal symmetry, and shoulder and hip alignment to optimize implant selection and surgical strategy [14].

During the physical examination, the medial and caudal attachments of the pectoralis major muscle were assessed while the patient actively contracted the muscle. To enhance intraoperative precision and ensure optimal symmetry, the anatomical relationship between the pectoralis major, its medial and caudal borders, and the inframammary fold was meticulously defined [13].

### Marking

Marking was performed with the patient in an upright position to ensure anatomical accuracy. Patients were instructed to stand with their weight evenly distributed on both feet, engage the abdominal muscles, maintain a posterior pelvic tilt, relax the shoulders, and allow the arms to hang naturally at their sides. This standardized posture facilitated precise and consistent preoperative markings, optimizing symmetry and surgical planning.

### Implant Selection

Implant selection is guided by a shared clinical decision-making process, incorporating both patient preferences and the surgeon’s expertise while being tailored to the patient’s unique anatomical characteristics [15–22].

The patient’s anatomy remains the most critical factor in determining the appropriate implant. Key anatomical considerations include height, weight, breast parenchymal thickness, and inframammary fold positioning. The goal is to select an implant that minimizes disruption to the natural fold position. Implant recommendations are typically provided within a predefined size range, allowing for individualized selection in collaboration with the patient.

In addition to anatomical factors, patient expectations and aesthetic preferences play a crucial role in implant selection. Patients' experiences within their social environment, as well as reference images of desired breast aesthetics, are carefully considered to align surgical outcomes with their expectations.

### Surgical Technique

The patient is positioned in the supine position with the arms secured at 90°, and the back is elevated by 5°–10° to optimize surgical exposure. The operative field is meticulously prepared using iodine-based antiseptics, and the nipple–areola complexes are protected with transparent adhesive strips to prevent distortion.

**Fig. 1:**
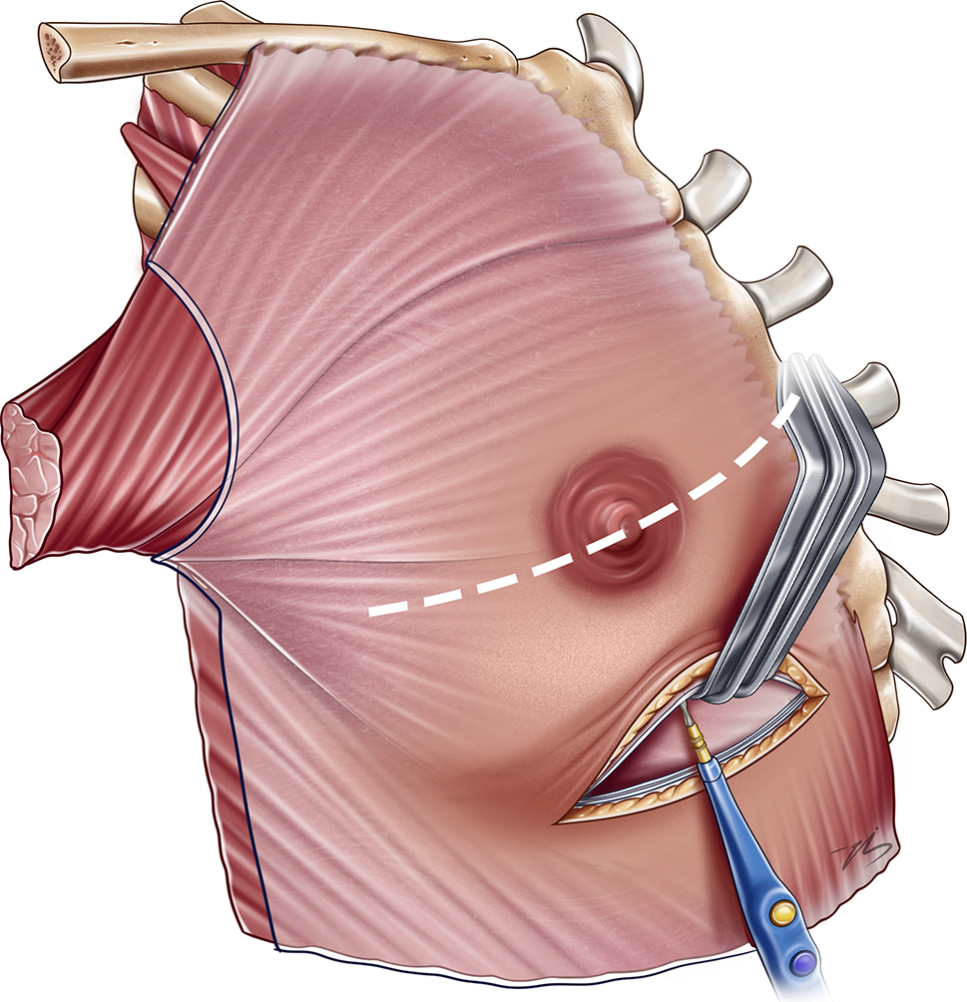
The fascia of the pectoralis major is dissected using cautery up to the superior border of the fourth rib (subfascial dissection border is indicated by the white dotted line)

An inframammary incision is made, followed by dissection through the dermis, subcutaneous fat, and superficial fascia. Subfascial dissection is performed using electrocautery up to the superior border of the fourth rib (Fig. 1). At this level, the pectoralis major muscle is transected in a full-thickness fashion, following a slightly inferolateral oblique direction toward the lateral confluence of the pectoralis major and minor muscles. The muscle is then elevated to the desired level, typically extending to the inferior border of the second rib.

Next, the caudal flap dissection is initiated. Using electrocautery, the flap is carefully separated from the rib periosteum. Vessels emerging from the fifth intercostal space are meticulously cauterized with bipolar electrocautery to minimize bleeding. The caudal flap extends to the inferior border of the fifth rib or the superior border of the sixth rib, with intraoperative modifications made as needed to accommodate implant dimensions. At this stage, the caudal attachments of the pectoralis major are incorporated into the flap, integrating the rectus abdominis fascia medially and, in some cases, the rectus abdominis muscle. Laterally, the flap includes the external oblique and serratus anterior fascia and muscles.

**Fig. 2:**
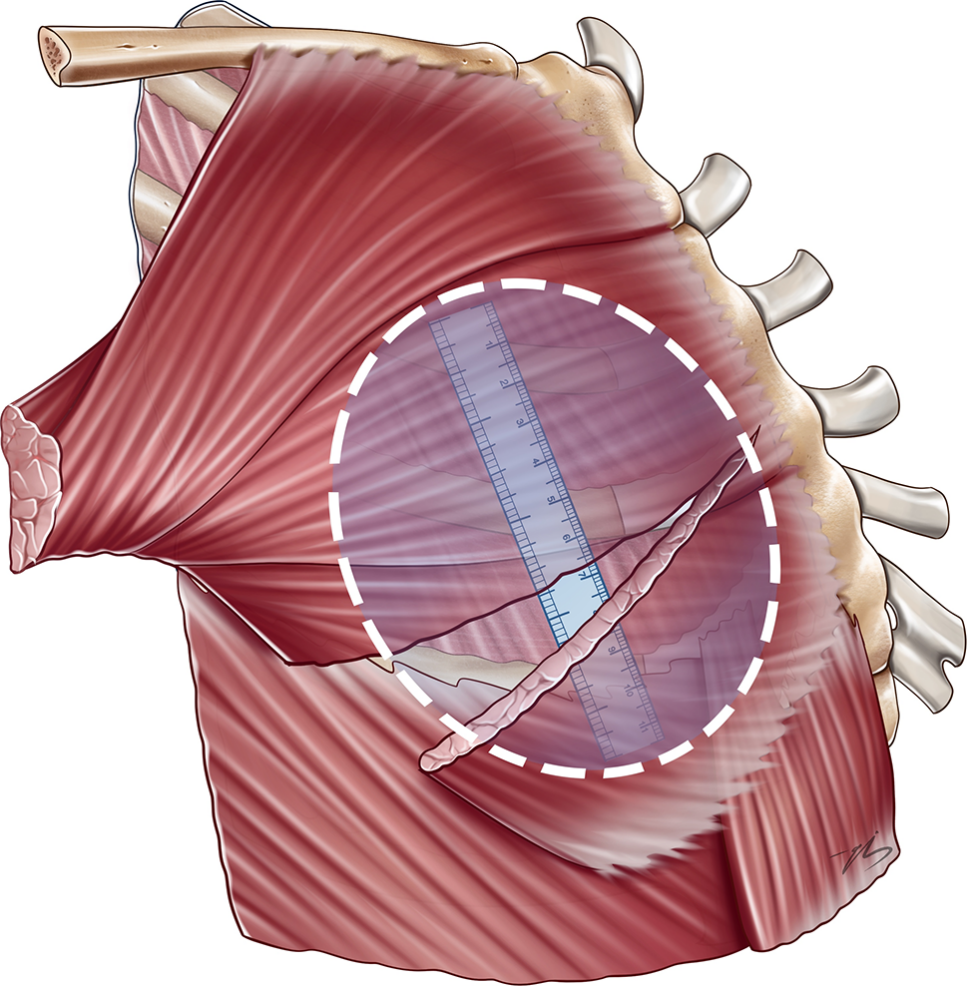
The white dotted circular lines show the implant pocket dissection area. The pocket size is measured with a paper ruler precut to the implant’s base width

Once the initial pocket dissection is completed, a precut paper ruler matching the implant’s base diameter is used to meticulously define the superior and inferior pocket borders (Fig. 2). Medial dissection preserves the medial attachments of the pectoralis major, while lateral dissection maintains the anatomical integrity of the confluence between the pectoralis major, pectoralis minor, and serratus anterior muscles. The implant is then securely positioned within the defined pocket, resembling a chestnut (“the implant”) emerging from its shell (“the superior and inferior flaps”)—an analogy that inspired the technique’s nomenclature.

Proper implant coverage by the flaps is confirmed through finger palpation. In cases where the caudal flap exhibits excessive tension, a single midline incision within the flap effectively relieves tightness and facilitates optimal implant positioning.

**Fig. 3:**
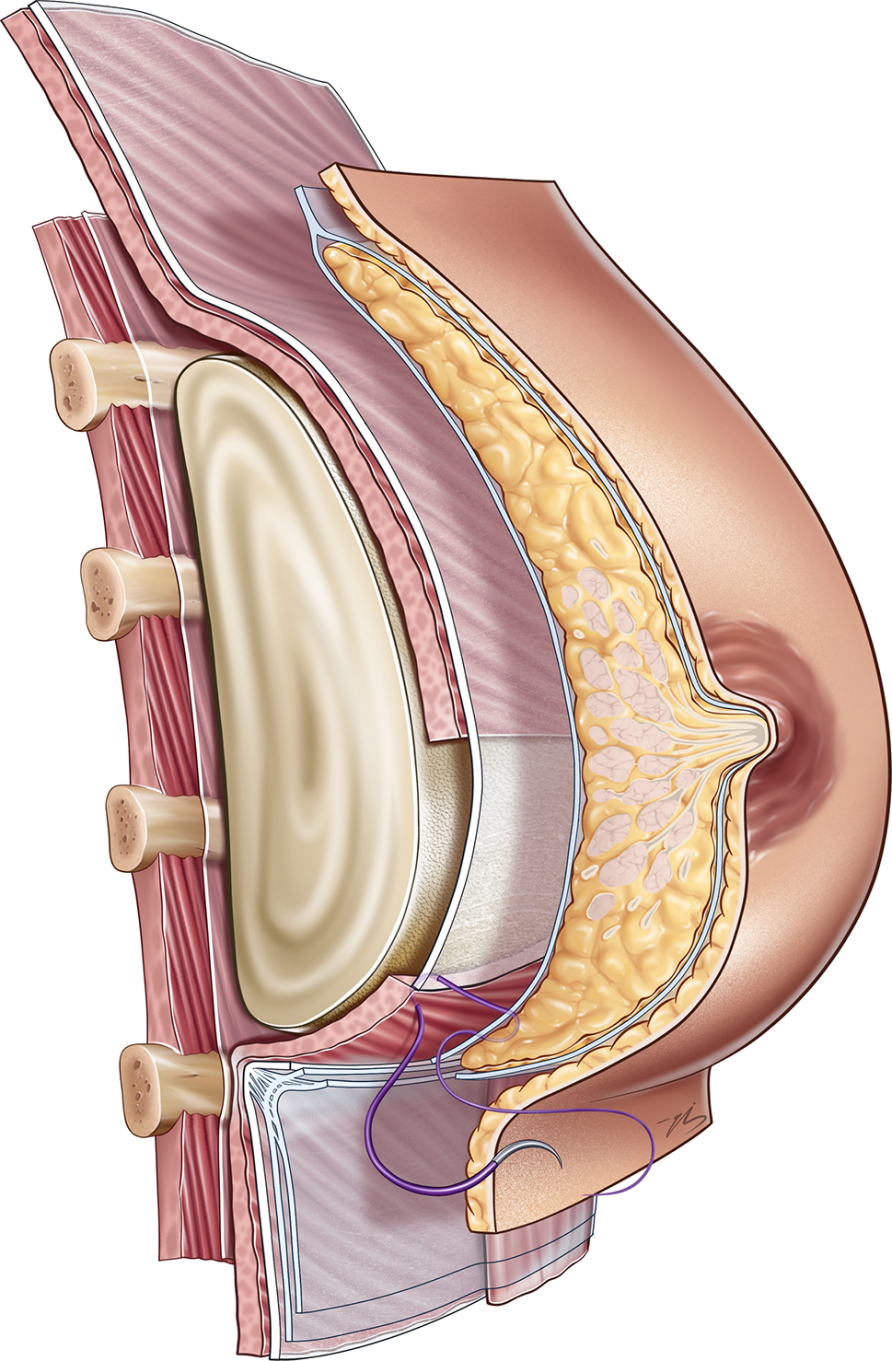
The chestnut technique. The implant is positioned behind a cranial flap superiorly, the pectoralis fascia at its midsection, and a caudal flap inferiorly. The pectoralis fascia is sutured to the caudal flap using 2/0 polyglactin 910 sutures

**Fig. 4:**
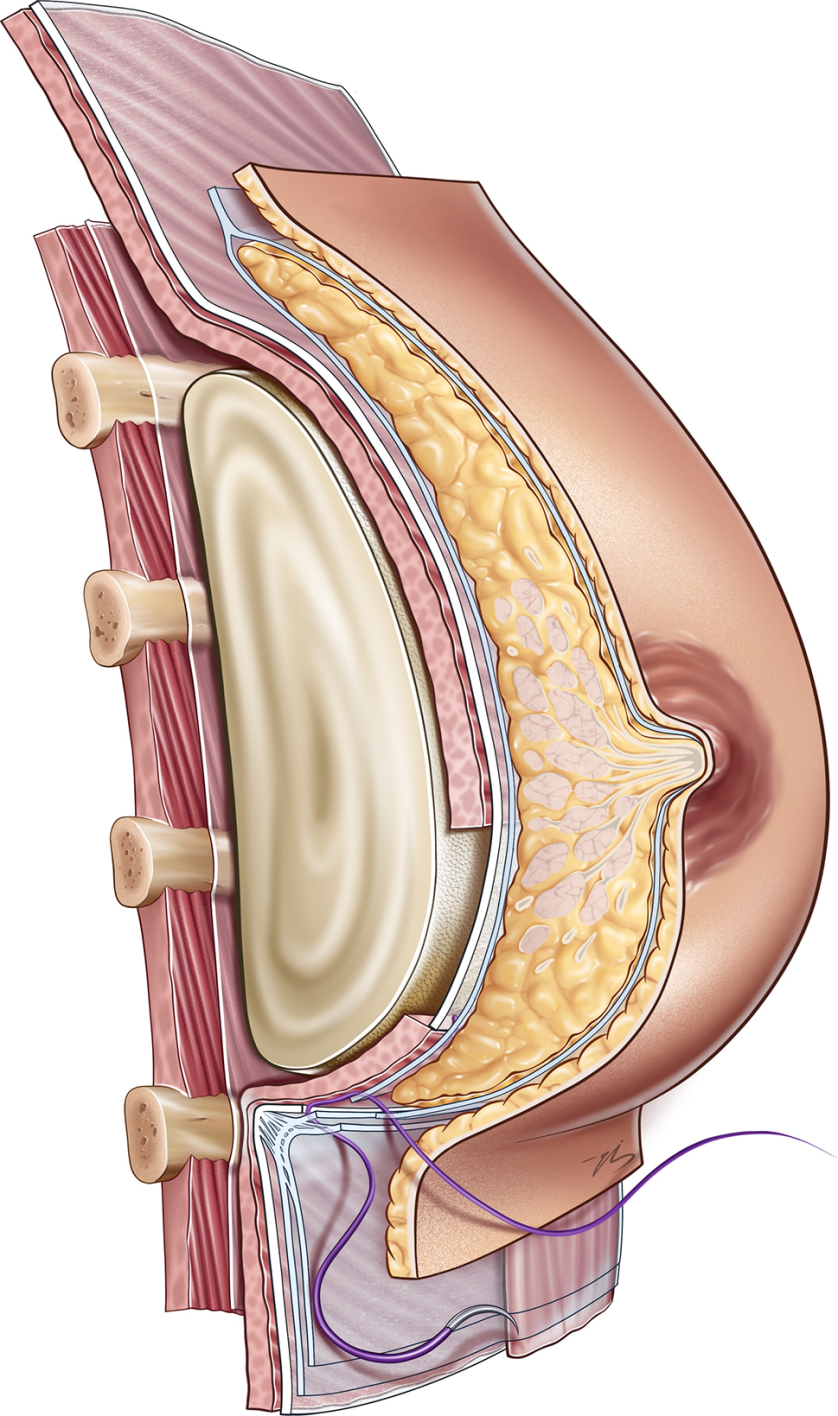
Suturing of the superficial fascia with 3/0 Vicryl to ensure fold stability

The pectoralis fascia forms the inferior border of the cranial flap and is sutured to the caudal flap using 2/0 polyglactin 910 sutures (Fig. 3). Additional reinforcement is achieved by approximating the superficial fascia above and below the incision with 3/0 polyglactin 910 sutures (Fig. 4). The skin is meticulously closed using a running 4/0 poliglecaprone 25 suture. Routine drain placement was not utilized, as meticulous hemostasis during caudal flap dissection and pocket preparation minimized the risk of seroma or hematoma formation. The surgical steps of the chestnut technique are demonstrated in the video.

### Postoperative Follow-up and Evaluation of Patient Satisfaction

Postoperative follow-ups were scheduled on postoperative day 1, at 1 week, 2 weeks, 1 month, 6 months, and 1 year. After this period, patients were advised to attend annual check-ups. During each visit, standardized photographs were taken, and patient feedback was systematically collected.

Patients were allowed to shower starting from postoperative day 3. By the end of the first week, strip dressings over the incision lines were replaced. A non-wired supportive bra was recommended for the first 6 weeks, during which patients were advised to avoid strenuous physical activities. In subsequent follow-ups, patients were questioned regarding their level of physical activity and any discomfort or complaints experienced during sports or exercise.

To assess patient satisfaction and health-related quality of life following breast augmentation, the BREAST-Q Augmentation Module was utilized [23]. Patients completed the BREAST-Q questionnaire preoperatively and at 1 year postoperatively, and their responses were converted into a 0–100 scale using the BREAST-Q scoring system, with higher scores indicating better patient-reported outcomes.

Of the 60 patients initially included, all patients completed the preoperative BREAST-Q and the postoperative 1-year follow-up and the BREAST-Q assessments. No patients were lost to follow-up during the first year, ensuring comprehensive data collection for outcome evaluation.

Assessments were performed by an independent physician who was not involved in the surgical procedures.

### Statistical Analysis

Statistical analyses were performed using SPSS version 29.0 (IBM SPSS Inc., Armonk, NY, USA). Continuous variables were expressed as mean ± standard deviation (SD). Preoperative and 1-year postoperative BREAST-Q scores were compared using paired t tests, as the data were confirmed to be normally distributed. A *p*-value of <0.05 was considered statistically significant.

Additionally, percentage changes were calculated to quantify improvements in patient-reported satisfaction and quality of life following breast augmentation.

## Results

### Demographic and Implant Data

A total of 60 patients underwent breast augmentation, with a mean age of 31.5 years at the time of surgery (range 18–50 years). The mean follow-up period was 31.8 months (range 12–56 months).

Mentor® (Johnson & Johnson MedTech, CA, USA) implants were used in 31 patients, while Motiva® (Establishment Labs, Costa Rica) implants were used in 29 patients. The mean implant size was 335 ± 48.55 cc (range: 235–450 cc). Demographic data and the subtypes of the implants used are presented in Table 1.

**Table 1:** Demographic and implant data

Parameter	Mean (+range)
Age at surgery	31.5 years (18–50)
Follow-up period	31.8 months (12–56)
Implant size	335 ± 48.55 cc (235–450)
Implant distribution*	SHPX-285 cc (*n*=5), SHPX-355 cc (*n*=5) RSF-335 cc (*n*=4), MPX-295 cc (*n*=4) SHPX-335 cc (*n*=3), TMPX-325 cc (*n*=3) RSF-425 cc (*n*=3), RSF-315 cc (*n*=2) RSF-295 cc (*n*=2), ERSF-295 cc (*n*=2) ERSF-450 cc (*n*=2), SHPX-380 cc (*n*=1) RSF-355 cc (*n*=1), CPG-333 330 cc (*n*=1) RSF-275 cc (*n*=1), THPX-340 cc (*n*=1) SHPX-415 cc (*n*=1), THPX-325 cc (*n*=1) CPG-323 300 cc (*n*=1), RSF-400 cc (*n*=1) THPX-325 cc (*n*=1); RSF-355 cc (*n*=1) ASFF-325 cc (*n*=1), RSF-255 cc (*n*=1), ERSF-315Q cc (*n*=1), RSF-235 cc (*n*=1), RSF-275 cc (n=1), CPG-323-300 cc (n=1) RSF-335 cc (n=1), ASMF46Q-320 cc(n=1) RSC-350 cc (n=1), RSF-450 cc (n=1) ERSF-335 cc (n=1), TMPX-405 cc (n=1) THPX-365 cc (n=1), TMPX-350 cc (n=1)

^*^The column represents the classification codes assigned by manufacturers for the implant brands and models used in patients. These codes primarily consist of letters and numbers, with the last three digits indicating the implant size in cubic centimeters (cm^3^).

Motiva® (Establishment Labs, Costa Rica): Implants from this brand are identified by the following codes: ERSF, ASFF, ASMF, RSF, and RSC.

Mentor® (Johnson & Johnson MedTech, CA, USA): Implants from this brand are identified by the following codes: SHPX, THPX, CPG-333, CPG-323, and TMPX.

### Complications

Postoperative complications were observed in 3 out of 60 patients (5%). One patient developed a subcutaneous hematoma on postoperative day 3, while two patients experienced localized seroma discharge through the incision site at postoperative weeks 2 and 4, respectively.

**Fig. 5:**
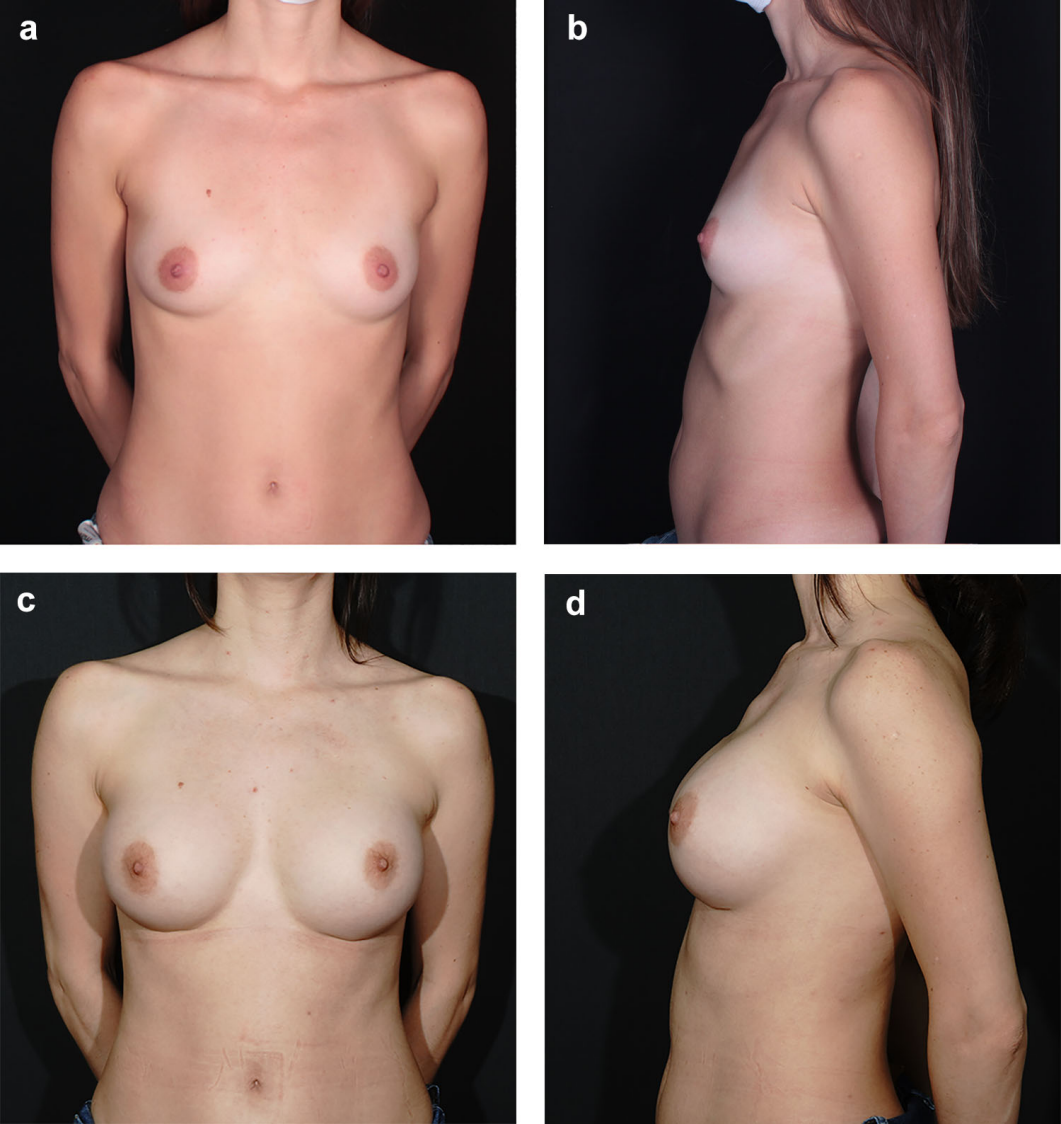
Preoperative **a** Antero-posterior (AP) and **b** Lateral views of a patient who underwent surgery using the chestnut technique; postoperative views at 38 months **c** AP and **d** Lateral. Lower pole stability is evident at the 3-year follow-up

**Fig. 6:**
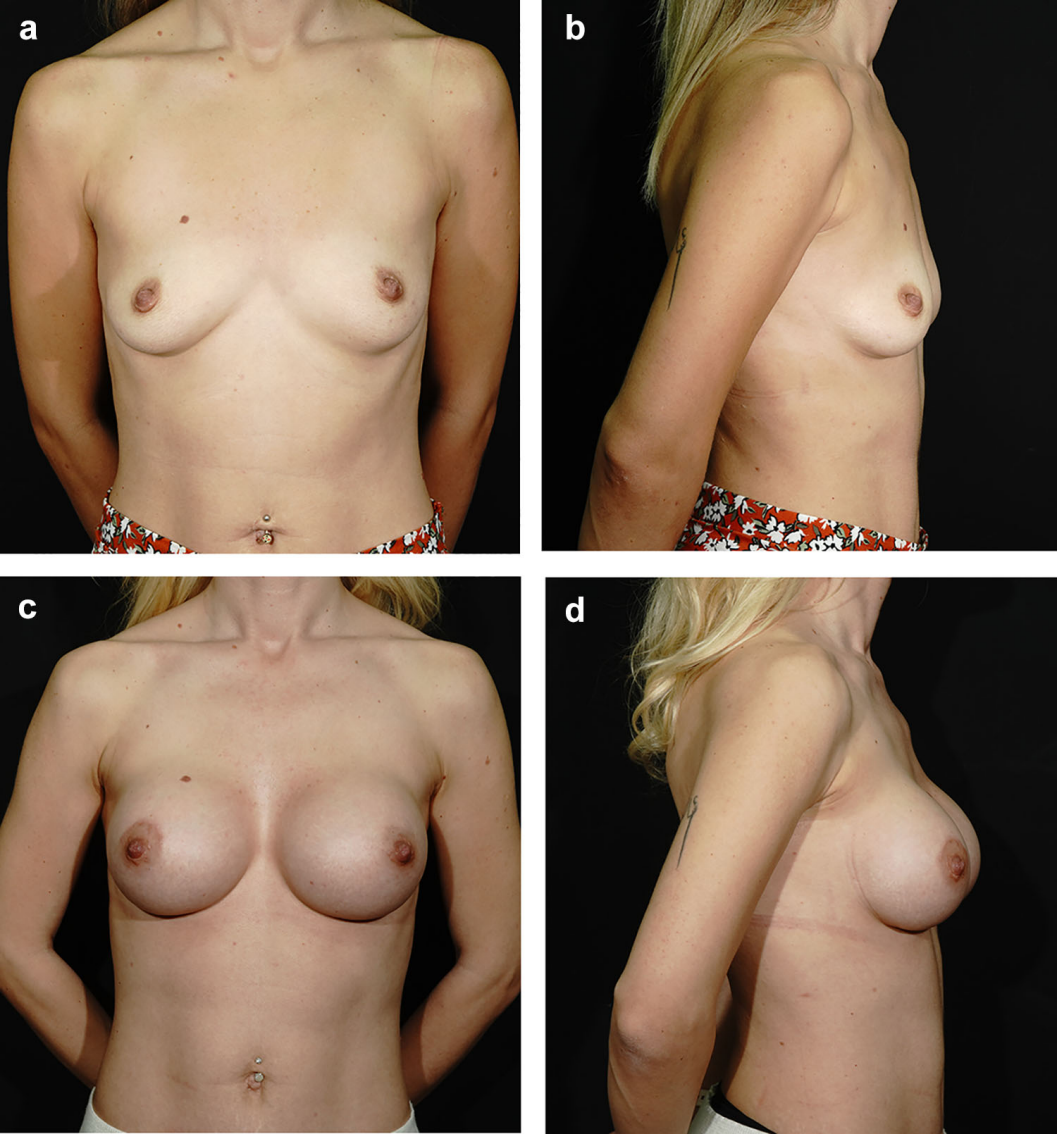
Preoperative **a** Antero-posterior (AP) and **b** Lateral views of a patient who underwent surgery using the chestnut technique; postoperative views at 22 months **c** AP and **d** Lateral. No complications were encountered in the patient at the 22nd month

All cases were successfully managed with local drainage, with no need for further intervention. During the follow-up period, no cases of implant malposition, animation deformity, or rippling were observed. Additionally, no clinically significant complications related to implant palpability were reported (Figs. 5 and 6).

### Postoperative Patient Satisfaction

The mean scores for each BREAST-Q domain are presented in Table 2. The preoperative mean score for satisfaction with breasts was 23.44 ± 10.20, which significantly increased to 84.46 ± 9.90 at 1 year postoperatively (*p* <0.001). This represents an improvement of +260.4%, indicating a substantial increase in patient satisfaction following surgery.

**Table 2:** Evaluation of patient satisfaction with using BREAST-Q scale

BREAST-Q Scale	Preop Mean	Preop SD	Postop Mean	Postop SD	Mean Change	% Change	*p*-value
Satisfaction with breasts	23.44	10.2	84.46	9.9	61.03	260.4	*p*<0.001
Psychosocial well-being	46.06	10.52	82.8	12.34	36.74	79.76	*p*<0.001
Sexual well-being	40.97	10.38	81.56	10.56	40.6	99.1	*p*<0.001
Physical well-being	75.47	4.65	75.78	5.68	0.31	0.4	0.75739

The mean psychosocial well-being score increased from 46.06 ± 10.52 preoperatively to 82.80 ± 12.34 postoperatively, reflecting a +79.8% improvement (*p* < 0.001). Patients reported feeling significantly more confident and comfortable with their body image after augmentation.

Patients also experienced significant improvements in sexual well-being, with scores rising from 40.97 ± 10.38 preoperatively to 81.56 ± 10.56 postoperatively (*p* < 0.001), corresponding to a +99.1% increase.

Unlike the other domains, physical well-being scores remained stable, with a preoperative mean of 75.47 ± 4.65 and a postoperative mean of 75.78 ± 5.68 (*p* = 0.757), indicating no statistically significant change.

## Discussion

Over the years, continuous advancements in implant technology and surgical techniques have contributed to improved aesthetic outcomes and reduced complication rates, particularly in terms of capsular contracture, infection, and implant malposition [5–7]. However, despite these refinements, achieving long-term implant stability remains a significant challenge, especially in patients with inadequate soft tissue support [6–11].

Among the key concerns in breast augmentation, inferior pole stability is crucial for ensuring long-term success [24–27]. Traditional augmentation techniques, including dual-plane and subfascial approaches, often fail to provide sufficient structural reinforcement, particularly in patients with thin breast tissue or weak inframammary fold integrity. This deficiency can result in complications such as implant displacement, bottoming-out, double-bubble deformity, rippling, animation deformity, and asymmetry. Various modifications have been introduced to optimize implant positioning and reduce these complications [28–32]. However, many conventional techniques do not offer a standardized and reproducible solution to these structural limitations. This ongoing challenge highlights the necessity for more advanced techniques to enhance implant stability and maintain long-term aesthetic outcomes.

Although shared clinical decision-making plays a role in implant selection, a patient’s anatomical features remain the primary determining factor. Numerous studies have evaluated different approaches to implant selection, with some adhering to industry standards and others employing tissue-based planning [16–22]. However, in practical applications, these studies yield comparable early postoperative results. Every experienced surgeon understands that the most critical factor affecting long-term outcomes is the passage of time and the morphological changes that occur during this period, including weight fluctuations, breastfeeding, and tissue alterations influenced by the implant’s weight and friction.

The chestnut technique was developed to address these challenges by mitigating the effects of implant weight and frictional forces through the caudal flap, offering a distinct advantage over other techniques from a long-term perspective. The medial and lateral boundaries of dissection in this technique are anatomically fixed, ensuring that the implant’s base fits precisely between the sternal attachments of the pectoralis major muscle medially and the anterior axillary line laterally. The inferior and superior boundaries are carefully marked based on the implant’s profile and shape characteristics. Patient-specific variations primarily involve the level of elevation of the caudal flap. When using a round implant, its base should align precisely with the intersection of the nipple meridian and equator, while for anatomical implants, the nipple–areola complex should correspond to the implant’s most projected point when held horizontally. This necessitates a more restricted caudal dissection when anatomical implants are used.

The chestnut technique modifies the dual-plane, triple-plane, and subfascial approaches by elevating the pectoralis major muscle from a more cranial point and integrating the pectoralis fascia into the cranial flap design [6, 7, 28]. The primary advantage of this technique is its ability to combine the static support of the caudal flap with the dynamic adaptability of the cranial flap, effectively preventing implant displacement, bottoming-out, animation deformity, double-bubble deformity, and disruption of the inframammary fold’s three-dimensional collagen structure. By employing a multilayered approach that integrates multiple fascial and muscle layers, this technique enhances implant stability and improves aesthetic outcomes.

The cranial flap is elevated with an incision starting at the superior border of the fourth rib, making it shorter than in dual-plane or total submuscular techniques. The cranial flap typically extends only above the implant’s midline. In contrast, in conventional dual-plane or full submuscular techniques, the implant is predominantly covered by the pectoralis major muscle, increasing the risk of superior displacement or animation deformity, particularly in patients with strong pectoral muscles [33, 34]. Another complication arising from excessive implant coverage by the pectoral muscle, particularly in patients with loose skin and breast tissue, is waterfall deformity. The literature suggests that this deformity can be mitigated by technique variations, such as the median cut, which reduces contact and shifts the center of gravity of the pectoral muscle covering the implant [35]. In the chestnut technique, the pectoral muscle is elevated more cranially, addressing the shortcomings in the design of the dual-plane technique. Although contradictory views exist in the literature, notably, no clinical difference in power was observed between transecting the muscle at this level and lower levels [36].

The cranial flap is almost conceptually identical to the cranial flap in the triple-plane technique. However, in practical terms, there are notable differences. In the triple-plane technique, the cranial flap lacks continuity with the pectoral fascia. Additionally, the elevation technique is performed in the opposite direction of the chestnut technique, with dissection proceeding from lateral to medial following a periareolar incision and dividing the breast tissue [28]. In the chestnut technique, special attention is given to preserving the integrity of the confluence formed by the pectoralis major and minor muscles and the serratus anterior muscle and fascia, approximately at the level of the anterior axillary line. The author believes that postoperative lateral malposition is likely associated with damage to this confluence. Therefore, we believe that the pectoral muscle flap should be dissected in a mediolateral fashion. Moreover, when examining the elasticity within the breast quadrants, it has been demonstrated that the lateral breast quadrant has a structure that is significantly prone to stretching or relaxation [37].

The pectoral fascia serves as an effective protective and covering layer [6]; however, its strength and thickness are, of course, not comparable to those of the pectoral muscles [38]. The soft implant, under the firm pressure of the cranial and caudal flaps, slightly protrudes toward the surface at its most prominent central point. In the chestnut technique, regardless of whether the implant is round or anatomical, its most prominent point is positioned and protruded directly beneath this fascia, which aligns with the level of the nipple–areola complex. This variable pressure applied to the implant ensures that it provides support precisely where the breast aesthetically requires the most projection—directly behind the nipple–areola complex.

A key consideration in planning the implant pocket in our technique is ensuring that the implant is perfectly centered within the breast footprint. While this does not initially conform to the generally accepted aesthetic proportions in breast aesthetics during the early postoperative period, the effects of gravity and tissue stretching gradually shift the initial 1:1 ratio toward the accepted standard. This transition results in the nipple position moving slightly upward, aligning with the 9:11 ratio within the breast hemisphere [39, 40].

The caudal flap is perhaps the most fundamental element of the chestnut technique. It supports the implant and preserves the structural integrity of the inframammary fold. Silicone implants are inherently heavy. Over time, gravitational forces combined with dynamic activities, such as running or jumping, can stretch the breast tissue, compromising its support [41].

The inframammary fold is a complex three-dimensional structure that exhibits significant anatomical and structural variability among individuals [42, 43]. Prolonged exposure to implant weight and frictional forces can compromise its integrity, leading to undesirable outcomes such as bottoming-out, implant slippage, or double-bubble deformity. The chestnut technique addresses these concerns by employing the caudal flap, which consists of the caudal portion of the pectoralis major, rectus abdominis, serratus anterior, and external oblique muscles, along with their associated fascial layers. This flap functions as a durable static support, effectively acting as a hammock that stabilizes the implant within the pocket. By suturing the caudal flap to the pectoral fascia, the continuity of implant coverage is ensured, preventing excessive lower pole expansion and maintaining the natural curvature of the inframammary fold.

Additionally, the caudal flap contributes to an added layer of coverage over the incision site, reinforcing implant stability and functioning similarly to an acellular dermal matrix [44, 45]. This approach preserves the three-dimensional fascial and collagen network of the inframammary fold while reducing the likelihood of long-term complications, such as implant migration or malposition. Unlike other augmentation techniques—including dual-plane, subfascial, subglandular, or full submuscular approaches—the chestnut technique uniquely incorporates this caudal flap as an intrinsic component of its design. While some publications describe muscle sling techniques or myofascial supportive flaps (MFF) to reinforce implant stability, the caudal flap in the chestnut technique closely resembles the "balcony" component of the triple-plane approach [29, 46].

In the triple-plane technique, following pectoral muscle transection, the creation of the caudal flap involves a dissection vector directed inferiorly from the nipple. However, due to this distance, achieving precise dissection at the desired millimetric level is technically challenging. While some argue that absolute control of the inframammary fold is impossible, the structured design of the caudal flap enhances confidence in maintaining fold stability and security [25]. Furthermore, the integrity of the lateral confluence of the pectoral muscles is more easily preserved with the chestnut technique compared to alternative methods. Additionally, the effectiveness of hemostasis in managing perforator vessels originating from the Würinger band is significantly improved when using an inframammary incision [47]. Our technique utilizes the inframammary incision, whose advantages over other incision sites have been extensively discussed in the literature. We believe that the periareolar incision should not be preferred in primary breast augmentation cases without a specific indication [48, 49].

The findings of our study demonstrate that the chestnut technique significantly enhances long-term results with high patient satisfaction with breast aesthetics, psychosocial well-being, and sexual well-being. These results underscore the positive impact of breast augmentation on overall quality of life, further reinforcing its role as a highly effective procedure for individuals seeking both aesthetic and psychological benefits. By integrating and optimizing the use of local muscle and fascial structures, the chestnut technique offers a reliable and structurally sound approach to breast augmentation. Its ability to position the implant as close as possible to the body’s center of gravity helps minimize the mechanical strain on the inframammary fold, reducing the risk of implant-related complications. This makes the technique particularly advantageous for patients with weak inframammary fold support, offering both enhanced stability and long-lasting aesthetic outcomes.

The chestnut technique has certain inherent limitations. First, the anatomical constraints of the submuscular plane restrict the available space between the origins and insertions of the involved muscles. While minor adjustments to implant shape and profile can allow for some volume variations, this technique is not suitable for large-volume implants. This aligns with the principle of *primum non nocere*, reinforcing the surgeon’s preference for moderate-sized implants and leading to the abandonment of the dual-plane technique in their practice. For patients specifically requesting this approach or opting for larger implants, the subfascial plane may be a more viable alternative, particularly for international patients with limited postoperative follow-up availability. Second, the cranial flap is designed to provide stable coverage over at least the upper third of the implant. However, in high-profile implants, such as ultra-high or corset-shaped designs, the cranial flap may not fully maintain its position, increasing the risk of implant exposure. To prevent this, implants with extreme profiles should be avoided when using this technique. Third, a potential limitation of submuscular and subfascial breast augmentation techniques is the risk of inferior migration of the breast parenchyma over the implant, leading to waterfall or snoopy deformity. Although no patients in our cohort demonstrated such complications during the follow-up period, the possibility remains in longer-term follow-up, particularly following significant weight fluctuations or pregnancy. The inherent mobility between the pectoral fascia and breast tissue necessitates consideration of this potential outcome [50]. Fourth, localized hematoma and seroma formation may occur due to bleeding beneath the caudal flap. In this study, three patients developed such complications within the first postoperative month. All cases were successfully managed with outpatient drainage without requiring further surgical intervention. Special attention must be given to meticulous hemostasis, particularly during caudal flap elevation, to prevent vascular bleeding, especially from the Würinger band. Future large-scale studies with long-term follow-ups and multi-surgeon involvement are necessary to fully establish the clinical utility, safety, and long-term benefits of the chestnut technique.

Fifth, although two different implant brands (Mentor® and Motiva®) were used in this cohort, no subgroup analysis was conducted to compare outcomes between them. Given the relatively small sample size for each implant type, any statistical comparison would lack sufficient power and could potentially lead to misleading interpretations. More importantly, the chestnut technique was designed to provide consistent structural support regardless of implant shape, texture, or brand. In our experience, this technique has demonstrated reproducible outcomes across varying implant profiles and manufacturers, suggesting that its stabilizing effect is primarily related to the surgical design rather than the prosthesis itself. Future large-scale studies may further explore implant-specific differences, but our preliminary findings support the robustness of the technique across implant variations.

Sixth, a notable limitation is the lack of a concurrent control group, which restricts the ability to draw comparative conclusions regarding complication rates or superiority over other techniques. Consequently, the findings should be interpreted within the context of a descriptive case series. Additionally, the objective validation of our study’s perceived superiority, particularly concerning complication rates, is impeded. This is largely attributed to the paucity of explicit documentation of complications such as rippling, double-bubble, bottoming-out, and waterfall deformities, even within extensive cohort studies involving 100,000 participants. As a result, readers are compelled to speculate that these specific complications are implicitly categorized under the broader classification of "re-operation" complications [51].

## Conclusion

The chestnut technique provides a safe and effective approach to primary breast augmentation, particularly in cases where enhanced soft tissue support is desired. By optimizing implant stability and minimizing displacement-related complications, it serves as a reliable alternative to conventional methods while ensuring predictable and aesthetically favorable outcomes.
